# miR-24-3p and Body Mass Index as Type 2 Diabetes Risk Factors in Spanish Women 15 Years after Gestational Diabetes Mellitus Diagnosis

**DOI:** 10.3390/ijms24021152

**Published:** 2023-01-06

**Authors:** Jessica Ares Blanco, Carmen Lambert, Manuel Fernandez-Sanjurjo, Paula Morales-Sanchez, Pedro Pujante, Paola Pinto-Hernández, Eduardo Iglesias-Gutiérrez, Edelmiro Menendez Torre, Elias Delgado

**Affiliations:** 1 Endocrinology, Nutrition, Diabetes and Obesity Group, Health Research Institute of the Principality of Asturias (ISPA), 33011 Oviedo, Spain; 2 Endocrinology and Nutrition Department, Asturias Central University Hospital, Av. Roma s/n, 33011 Oviedo, Spain; 3 Medicine Department, University of Oviedo, 33011 Oviedo, Spain; 4 University of Barcelona, 08007 Barcelona, Spain; 5 Department of Functional Biology, University of Oviedo, 33007 Oviedo, Spain; 6 Translational Health Interventions Group, Health Research Institute of the Principality of Asturias (ISPA), 33011 Oviedo, Spain; 7 Centre for Biomedical Network Research on Rare Diseases (CIBERER), Instituto de Salud Carlos III, 28029 Madrid, Spain

**Keywords:** gestational diabetes mellitus, type 2 diabetes mellitus, body mass index, miR-24-3p

## Abstract

Gestational diabetes mellitus (GDM) is defined as any degree of glucose intolerance that is diagnosed for the first time during pregnancy. The objective of this study is to know the glucose tolerance status after 15 years of pregnancy in patients diagnosed with gestational diabetes and to assess the long-term effect of GDM on the circulating miRNA profile of these women. To answer these, 30 randomly selected women diagnosed with GDM during 2005–2006 were included in the study, and glucose tolerance was measured using the National Diabetes Data Group criteria. Additionally, four miRNAs (hsa-miR-1-3p, hsa-miR-24-3p, hsa-miR-329-3p, hsa-miR-543) were selected for their analysis in the plasma of women 15 years after the diagnosis of GDM. In our study we discovered that, fifteen years after the diagnosis of GDM, 50% of women have some degree of glucose intolerance directly related to body weight and body mass index during pregnancy. Dysglycemic women also showed a significantly increased level of circulating hsa-miR-24-3p. Thus, we can conclude that initial weight and BMI, together with circulating expression levels of hsa-miR-24-3p, could be good predictors of the future development of dysglycemia in women with a previous diagnosis of GDM.

## 1. Introduction

Gestational diabetes mellitus (GDM) refers to abnormal glucose tolerance, which is diagnosed during pregnancy and disappears postpartum. According to data from the most recent International Diabetes Federation Atlas (IDF, 10th edition, 2021), 16.7% of pregnant women present some form of hyperglycemia, and 80.3% are diagnosed with GDM [1]. Principal risk factors for the development of GDM are overweight/obesity, family history of dysglycemia, and advanced maternal age [2]. In fact, the prevalence of GDM increased to more than 30% in women older than 40 years and more than 40% in women older than 45 years [1,2].

It is well known that women who have been diagnosed with GDM are at higher risk for developing type 2 diabetes (T2D); specifically, compared to the general population, women with a personal history of GDM have up to 10-fold higher risk of developing T2D [3].

Although GDM is diagnosed when metabolic alterations are already established, it could also be identified as an opportunity to intervene and control these women in order to prevent the development of T2D [4,5]. In fact, modifiable lifestyle factors can be identified as unhealthy diet habits, sedentarism, or combined associations of both. We do not know if modifying these factors in GDM patients would reduce the risk of T2D, even among overweight/obese women [6].

There is increasing evidence that epigenetic processes, including non-coding RNAs, play a role in the development of metabolic diseases [7]. Numerous studies have proposed the use of circulating microRNAs (c-miRNAs) as diagnostic, prognostic, and therapeutic biomarkers of diverse pathological processes, including metabolic diseases [8,9,10]. In the last years, a considerable amount of information has been published describing the association of GDM with the expression of certain miRNAs detected in plasma and different tissues, as we have recently reviewed [11].

To assess the long-term effect of GDM on the epigenetic modulation mediated by c-miRNAs, a restricted miRNA profile was analyzed in women who were diagnosed with GDM 15 years ago, whether or not they eventually developed T2D or prediabetes. We also determined if these alterations were associated with obesity or overweight status during pregnancy and after 15 years.

This study is an opportunity to develop further investigations in epigenetics pathophysiology involved in the progression of GDM to T2D through glucose metabolism alterations.

## 2. Results

### 2.1. Glycemic Status in Women 15 Years after the Diagnosis of GDM

Among the 30 women included in the study, we found that, currently, half of them remained normoglycemic (n = 15, 50%), while the other half had some type of dysglycemia, one-third of them (n = 10, 33%) had prediabetes, and five presented type 2 diabetes (T2D; n = 5; 16.7%), only one previously diagnosed.

When we classified these women regarding their glycemic status, we observed that those classified as dysglycemic had a significantly higher BMI during pregnancy than those classified as normoglycemic. Interestingly, 15 years after pregnancy, those women still showed a significantly increased BMI, as well as greater waist perimeter and lean and fat mass. Not only body mass composition and glucose status but also lipid status was altered. Specifically, dysglycemic women showed reduced HDL and increased TG levels compared to normoglycemic women (Table 1).

### 2.2. Clinical Predictors for the Diagnosis of T2D in Women 15 Years after the Diagnosis of GDM

**Figure 1:**
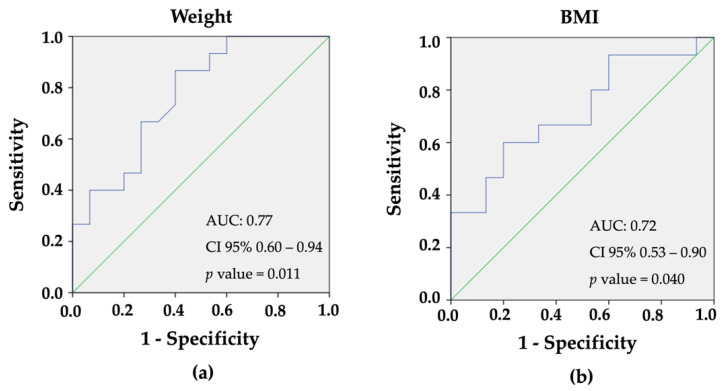
Receiver-Operating-Characteristic (ROC) curves for (**a**) weight (AUC = 0.77; *p* < 0.011) and (**b**) BMI (AUC = 0.72; *p* < 0.040) of women as predictors for the diagnosis of type 2 diabetes, 15 after the diagnosis of gestational diabetes.

The only variables presenting clinical relevance and statistical significance (*p* < 0.05) during pregnancy upon univariate analysis were weight and BMI (Table 2). ROC curves were generated to determine both their cut-off levels in order to predict further dysglycemia (Figure 1a,b). Women with increased weight and BMI were at risk of suffering from dysglycemia 15 years after GDM diagnosis, according to univariate analysis. The positive predictive value for developing dysglycemia for a weight higher than 69 kg was 77.0%, whereas, for a BMI higher than 26 kg/m^2^, it was 72.1%.

### 2.3. Plasma Circulating miRNA Expression Profile 15 Years after the Diagnosis of GDM

**Figure 2:**
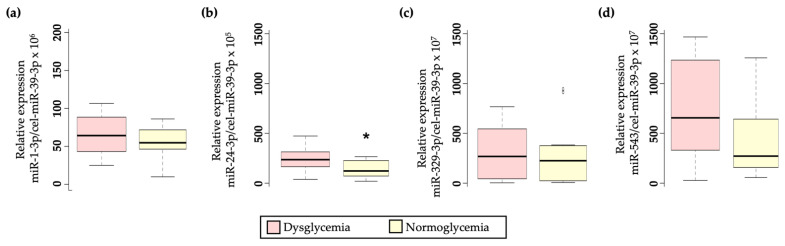
Boxplot diagram showing differential circulating miRNA expression profile in GDM women depending on their glycemic status after 15 years. (**a**) relative hsa-miR-1-3p expression, (*p* = 0.385); (**b**) relative hsa-miR-24-3p expression (*p* = 0.030); (**c**) relative hsa-miR-329-3p expression (*p* = 0.596); (**d**) relative hsa-miR-543 expression (*p* = 0.071). * *p*-value < 0.05 cel-miR-39-3p was used for miRNA expression normalization.

Four miRNAs were selected for their analysis in the plasma of women previously diagnosed with GDM based on literature [12,13,14,15,16]. Among them, only hsa-miR-24-3p showed a significant upregulation in dysglycemic women (*p* = 0.020; Figure 2b).

Additionally, although it does not reach significance, hsa-miR-543 showed the same trend as hsa-miR-24-3p, elevated in the plasma of dysglycemic women (*p* = 0.071; Figure 2d). No significant differences were observed in either hsa-miR-1-3p (Figure 2a) or hsa-miR-329-3p (Figure 2c).

**Figure 3:**
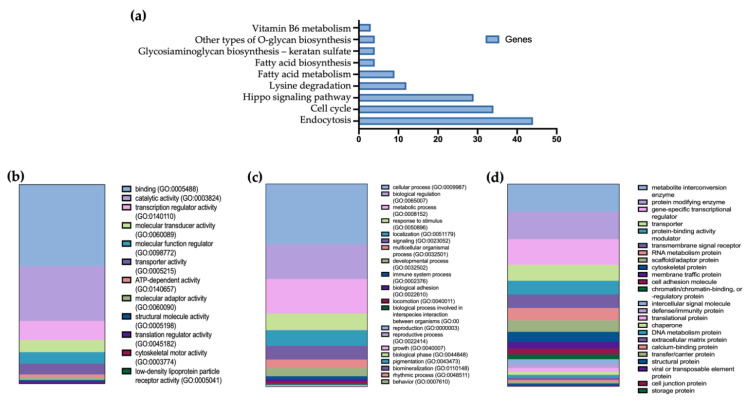
miR-24-3p and miR-543 enrichment pathway analysis. (**a**) Bar plot showing Kyoto Encyclopedia of Genes and Genomes (KEGG) pathway for all predicted target genes expressed as the number of genes per pathway. (**b**) Gene Onthology Molecular Processes, (**c**) Gene Onthology Biological Processes, and (**d**) Protein Classes affected by miRNAs target genes, and expressed as the number of target genes involved in each process.

Subsequently, bioinformatic tools were used to find the predicted target genes for both hsa-miR-24-3p and hsa-miR-543. Then, based on the Kyoto Encyclopedia of Genes and Genomes (KEGG), pathway analysis for all predicted target genes was finally performed. Excluding cancer-related terms, nine pathways were found to be affected by these miRNAs, including endocytosis, cell signaling, and fatty acid-related pathway (Figure 3a).

Additionally, Gene Ontology enrichment analysis was performed to investigate which specific molecular processes (Figure 3b), biological processes (Figure 3c), and protein classes (Figure 3d) are more likely to be associated with the miRNA target genes.

## 3. Discussion

According to data from the International Diabetes Federation, the incidence of T2D has risen significantly in the last years, becoming one of the biggest health problems of the 21st century, and affecting one in ten people in the world [1]. However, there are still many cases of T2D that are not diagnosed [17].

Different risk factors may predispose a person to suffer from T2D, including the previous development of GDM [3,18,19,20]. In fact, in this study, we found that half of the women suffering from GDM will develop any type of dysglycemia 15 years later. In addition, among the women with some type of dysglycemia, 33% were diagnosed with T2D, with only 20% having been previously diagnosed. These data reinforce the need to search for different biomarkers that can help identify those women who, having been diagnosed with GDM during pregnancy, are at higher risk of developing T2D.

It is well established that obesity and overweight are widely related to the development of both T2D [21] and GDM [22]; thus, it is not surprising that those women diagnosed with T2D present a significantly higher weight and BMI compared to non-T2D women. The measurement of BMI as an obesity predictor, although widely used, has been questioned on many occasions since it does not differentiate between fatty and non-fatty tissues (muscle, bone, etc.) [23]. Therefore, BMI measurement might be a poor approach to assess obesity in athletes whose body weight is highly influenced by muscle mass but not in middle-aged women with normal physical activity [23]. Nevertheless, in this study, in addition to BMI measurement, we decided to measure other factors such as waist circumference and fat and lean mass percentage, all of which were increased in the group of women with dysglycemia.

By performing a univariant analysis, we have discovered that the limiting factor that best predicts the future development of T2D in women with a previous diagnosis of GDM is the BMI during pregnancy, being those women with a BMI higher than 26 kg/m^2^ at increased risk of future development of dysglycemia. The short-and long-term consequences of gestational overweight have previously been described, increasing the risk of premature pregnancy loss, fetal malformations, and premature births, and also increasing the future cardiometabolic risk [24,25]. In this study, we have included hyperglycemic risk to the long-term consequences of pregnancy overweight, further reinforcing the need to establish awareness campaigns and strategies to prevent excessive weight gain in women during pregnancy.

Changes during pregnancy can also lead to epigenetic modifications that alter gene expression without affecting the DNA sequence. This epigenetic alteration includes non-coding RNA, such as miRNA modifications [26]. In this line, circulating miRNAs [27,28,29] have emerged in recent years as potential diagnostic and prognostic biomarkers for many diseases, including both GDM [30] and T2D [8]. Among the different miRNAs related to T2D [31,32], we have selected hsa-miR-24-3p, hsa-miR-543-3p, hsa-miR-329-3p, and hsa-miR-1-3p. Then we analyzed the circulating miRNA profile of women 15 years after the diagnosis of GDM; we observed that both, hsa-miR-24-3p and hsa-miR-543-3p, were increased in those women diagnosed with T2D, although only changes in hsa-miR-24-3p were significant. Joglekar et al. have previously identified hsa-miR-543-3p and hsa-miR-329-3p as T2D predictors in GDM women [12]; however, in our cohort, we have not been able to corroborate their results.

By contrast, hsa-miR-24-3p has not been deeply studied in the development of T2D, and only a few authors have related circulating levels of hsa-miR-24-3p with the diagnosis of diabetes. In addition, while some authors find lower levels of this miRNA in diabetic patients compared to controls [8,15,30], other studies state that metformin treatment and improvement in glycemic status in T2D patients are directly associated with decreased levels of hsa-miR-24-3p [33], it is important to note that those works in which the levels of hsa-miR-24-3p were reduced in patients with T2D, the patients were treated with different hypoglycemic drugs, so, as has already been shown with metformin, not only glucose and HbA1c levels could be affected, but also the expression levels of hsa-miR-24-3p.

Thus, it would be interesting to reassess the hsa-miR-24-3p levels of these women, recently diagnosed with IGT or T2D, once their glycemic levels are controlled to determine if they return to normal. Additionally, based on the bioinformatic approach, we could also relate the observed miRNA changes with fatty acid synthesis and metabolism, highlighting the importance of body composition in the development of T2D.

Many studies have described the relationship between the development of T2D in previously diagnosed GDM women; however, why some women develop T2D and others do not is not fully established. In this study, we highlight the importance of body composition for the development of T2D. Here, we propose that initial BMI, together with circulating expression levels of hsa-miR-24-3p, could be good predictors of the future development of dysglycemia in women with a previous diagnosis of GDM. Although the results obtained are of great importance, larger cohort and prospective studies should be carried out to establish BMI and the levels of circulating hsa-miR-24-3p as prognosis biomarkers for the development of T2D in pregnant women.

## 4. Materials and Methods

### 4.1. Participants

This is a retrospective cohort study that includes 30 randomly selected women who had been diagnosed with GDM during 2005–2006 at the Hospital Universitario Central de Asturias. All women studied belonged to a single ethnic group (white Caucasian). GDM was assessed by using the National Diabetes Data Group (NDDG) criteria after a 100 g oral glucose tolerance test (OGTT). GDM was diagnosed if two or more plasma glucose levels met or exceeded the following threshold: fasting glucose of 105 mg/dL, 190 mg/dL after 1 h, 165 mg/dL after 2 h, or 145 mg/dL after 3 h. Women were excluded if they had pre-existing diabetes, an abnormal result on a glucose screening test before 24 weeks of gestation, prior gestational diabetes or multifetal gestation; if they were taking corticosteroids; if there was a known fetal abnormally; or if imminent or preterm delivery was likely because of maternal disease or fetal conditions. Biochemical and anthropometric data were retrospectively obtained from the clinical history at the moment of GDM diagnosis. Informed consent was obtained from all volunteers, and the study protocol was approved by the HUCA ethical committee (Project identification code: CEImPA: 2020.056; acceptance date: 27 February 2020) that is consistent with the principles of the Declaration of Helsinki A complete clinical history was obtained, as well as body composition assessment using Bioelectrical Impedance Analysis technology (Tanita, T5896 Tokyo, Japan), which is a validated method to determine the total and segmental composition, anthropometric measurements, and a complete blood test was performed 15 years after the diagnosis of GDM.

### 4.2. Diagnosis of Type 2 Diabetes Mellitus

In 2020 (15 years after GDM diagnosis), all participants underwent a 75 g OGTT in order to analyze their actual glycemic profile. Women were classified into two groups: normoglycemic or dysglycemic, accordingly to ADA diagnostic criteria guidelines, as shown in Table 3 [34]. Additionally, dysglycemic women were subclassified as impaired glucose tolerance (IGT) and type 2 diabetes (T2D).

### 4.3. Blood Collection and Sample Preparation

Overnight fasting peripheral blood samples were collected from all subjects in EDTA-containing Vacutainer tubes (BD Biosciences, Franklin Lakes, NJ, USA). Blood samples were immediately centrifuged at 800× *g* for 15 min at 4 °C. The top layer containing the plasma was divided into aliquots and stored at −80 °C until further analysis.

### 4.4. miRNA Isolation and Quantification

For miRNA expression analysis, total RNA was isolated from 200 µL of frozen plasma samples in silica membrane columns using the miRNeasy Serum/Plasma Advanced Kit (Qiagen, Hildem, Germany) according to the manufacturer’s instructions. The mixture was supplemented with 1.5 µg of bacteriophage MS2 carrier RNA (Roche, Merck, Darmstadt, Germany) to improve isolation yield and a known amount of the synthetic *Caenorhabditis elegans* miR-39-3p (cel-miR-39-3p, Invitrogen, Waltham, MA, USA), lacking sequence homology to human miRNAs, was added as an external reference (1.6 × 10^8^ copies/μL). RNA was finally eluted into 20 μL of nuclease-free water and stored at −80 °C until further use.

Isolated total RNA was reverse transcribed into cDNA using the TaqMan advanced miRNA cDNA synthesis kit (Life Technologies, Carlsbad, CA, USA). miRNA expression analysis was carried out by quantitative PCR using TaqMan^®^ Gene Expression assays (Applied Biosystems, Waltham, MA, USA; Table 4) and the Applied Biosystems Prism 7900HT Sequence Detection System (Applied Biosystems, Waltham, MA, USA) according to manufacturer’s instructions. miRNA expression data were expressed as target miRNA expression relative to the corresponding housekeeping expression (ΔC_T_ = C_T_ [miRNA] − C_T_ [cel-miR-39-3p]) [35]. The relative expression of each miRNA was reported as 2^−ΔCT^.

### 4.5. Functional Enrichment Analysis

For each miRNA, experimentally validated targets were retrieved from the miRTarBase v.7 database [36]. Pathway annotations for each gene were retrieved from KEGG pathways using Diana mirpath v.3 [37]. Thus, we obtained gene sets and metabolic pathways linked to miRNA target genes. The results output a log odds ratio for each interrogated gene set, along with raw and false discovery adjusted p-values. The data represented a number of genes per pathway.

We used target mining analysis by miRWalk, which defined a complete list of gene targets. With this database, we used the Gene Ontology of molecular functions, biological processes, and protein classes on the Pantherdb 17.0 tool. All data represented were by the number of genes targeted.

### 4.6. Statistical Analysis

We divided the 30 participants into two groups depending on their altered glucose metabolism after 15 years of pregnancy. We recorded different biochemical and anthropometric parameters both at delivery and during pregnancy. The Shapiro-Wilk test was performed to assess normality. Then, differences between normoglycemic and dysglycemic women were analyzed with a non-parametric Mann-Whitney test. Data are expressed as median [max-min] unless stated.

Univariate analysis was performed first to determine associations between risk factors and further dysglycemia (prediabetes or T2D). A level of significance of *p* < 0.05 and 95% confidence intervals were adopted. Variables presenting clinical relevance and statistical significance (*p* < 0.05) upon univariate analysis were included in the logistic regression model. ROC curves were generated to determine cut-off values of BMI and weight in order to identify them as strong predictors of dysglycemia. Statistical analysis was performed using Statistical Package of Social Science (SPSS), version 21.0 for Windows and JASP software, version 0.14.1.

## 5. Conclusions

In summary, in the present study we have performed a follow up of previously diagnosed GDM women to asses their glycemic status fifteen years after pregnancy. We observed that 50% of women had developed some degree of glucose intolerance, which was directly related to body weight and body mass index during pregnancy. Additionally, we observed that dysglycemic women also showed a significantly increased level of circulating hsa-miR-24-3p. In conclusion, although more extensive studies should be performed, our results suggest that body composition during pregnancy, as well as circulating hsa-miR-24-3p measurement, could help to identify those women at higher risk of developing T2D, thus being able to implement different strategies that allow us to reduce the risk of women with a previous diagnosis of GDM to develop T2D.

## Figures and Tables

**Table 1:** Clinical characteristics of participants.

		Normoglycemya	Dysglycemia	
	N	15	15	
During Pregnancy	Weight (kg)	63.3 [52–83]	74.3 [59–108.9]	**0.011**
	BMI (kg/m^2^)	25.68 [21.45–31.83]	28.63 [11.06–39.05]	**0.04**
	100 g OGTT Basal	85 [75–100]	89 [74–104]	0.077
	100 g OGTT 1 h	202 [142–241]	203 [176–241]	0.43
	100 g OGTT 2 h	181 [166–210]	194 [149–270]	0.262
	100 g OGTT 3 h	141 [74–171]	155 [90–208]	0.11
15 years after pregnancy	Age	50 [42–61]	50 [44–58]	0.692
	HbA1c (%)	5.4 [4.9–5.7]	5.8 [5.1–6.8]	**<0.001**
	Weight (kg)	60.1 [53.2–87.7]	77.9 [58.5–131.3]	**0.008**
	BMI (kg/m^2^)	23.0 [20.5–34.1]	29.2 [22.1–46.0]	**0.007**
	Waist (cm)	80 [66–102]	95 [78–127]	**0.035**
	Lean mass (kg)	41.3 [35.2–49.1]	47.3 [40.5–63.9]	**0.007**
	Fat mass (kg)	16.4 [12.6–38.6]	29.4 [16.9–48.4]	**0.03**
	75 g OGTT Basal	85 [64–96]	95 [62–147]	**0.042**
	75 g OGTT 2 h	82 [29–143]	161.5 [40–238]	**<0.001**
	Total Ch (mg/dL)	202 [182–332]	215 [153–280]	0.999
	HDL-Ch (mg/dL)	64 [54–93]	48 [40–75]	**0.006**
	LDL-Ch (mg/dL)	113 [61–163]	120 [75–194]	0.407
	TAG (mg/dL)	86 [57–218]	135 [49–244]	**0.016**
	AST (mg/dL)	17 [10–54]	21 [10–91]	0.12
	ALT (mg/dL)	12.5 [7–26]	22 [8–80]	**0.008**
	GGT (mg/dL)	14 [8–24]	23 [11–84]	**0.003**

Participants are subdivided into two groups (Normoglycemic and dysglycemic) according to their glycemic status 15 years after pregnancy. Data expressed as median [range]. Significant changes are marked in bold. Significant measures marked in bold BMI = body mass index; OGTT = Oral Glucose Tolerance Test; HbAc1 = glycated hemoglobin; Ch = cholesterol; HDL = high-density lipoproteins; LDL = low-density cholesterol; TG = Triglycerides.

**Table 2:** Regression analysis showing weigh and BMI during pregnancy as an independent predictor of future development of T2D.

	Odds Ratio (OR)	95% CI	*p*-Value
Weight during pregnancy	1.66	1.06–2.60	0.04
BMI during pregnancy	0.29	0.08–0.99	0.04

Significant measures marked in bold. CI = Confidence interval; BMI = body mass index.

**Table 3:** Criteria for the diagnosis of Impaired glucose tolerance and type 2 diabetes.

	Normoglycemia	Dysglycemia	
		Impaired Glucose Tolerance	Type 2 Diabetes
Basal blood glucose (mg/dL)	<100	100–125	>126
HbA1c (%)	<5.7	5.7–6.4	>6.5
2-h OGTT blood glucose (mg/dL)	<140	140–200	>200

OGTT: Oral Glucose Tolerance Test; HbA1c = glycated haemoglobin.

**Table 4:** miRNAs sequence and expression profile.

miRNA	Reference	Sequence
hsa-miR-1-3p	477820_mir	5′-UGGAAUGUAAAGAAGUAUGUAU-3′
hsa-miR-24-3p	477992_mir	5′-UGGCUCAGUUCAGCAGGAACAG-3′
hsa-miR-329-3p	478029_mir	5′-AACACACCUGGUUAACCUCUUU-3′
hsa-miR-543	478155_mir	5′-AAACAUUCGCGGUGCACUUCUU-3′
cel-miR-39-3p	478293_mir	5′-UCACCGGGUGUAAAUCAGCUUG-3′

## Data Availability

Not applicable.
